# Decoy peptides effectively inhibit the binding of SARS-CoV-2 to ACE2 on oral epithelial cells

**DOI:** 10.1016/j.heliyon.2023.e22614

**Published:** 2023-11-20

**Authors:** Lai-Keng Loi, Cheng-Chieh Yang, Yu-Cheng Lin, Yee-Fun Su, Yi-Chen Juan, Yi-Hsin Chen, Hsiu-Chuan Chang

**Affiliations:** a Department of Dentistry, National Yang Ming Chiao Tung University, Taipei, Taiwan; b Department of Stomatology, Oral & Maxillofacial Surgery, Taipei Veterans General Hospital, Taipei, Taiwan; c iStat Biomedical Co., Ltd, New Taipei City, Taiwan; d Institute of Oral Biology, National Yang Ming Chiao Tung University, Taipei, Taiwan

**Keywords:** SARS-CoV-2, COVID-19, Spike protein, ACE2, Oral epithelial cell, Decoy peptide

## Abstract

The entry of SARS-CoV-2 into host cells involves the interaction between the viral spike protein and the human angiotensin-converting enzyme 2 (ACE2) receptor. Given that the spike protein evolves rapidly to evade host immunity, therapeutics that block ACE2 accessibility, such as spike decoys, could serve as an alternative strategy for attenuating viral infection. Here, we constructed a drug screening platform based on oral epithelial cells to rapidly identify peptides or compounds capable of blocking the spike-ACE2 interaction. We engineered short decoy peptides, 8 to 14 amino acids in length, using the spike protein's receptor-binding motif (RBM) and demonstrated that these peptides can effectively inhibit virus attachment to host cells. Additionally, we discovered that diminazene aceturate (DIZE), an ACE2 activator, similarly inhibited virus binding. Our research thus validates the potential of decoy peptides as a new therapeutic strategy against SARS-CoV-2 infections, opening avenues for further development and study.

## Highlights

• Engineered decoy peptides derived from the viral spike protein effectively inhibits spike-ACE2 interaction.
• Diminazene aceturate (DIZE), an ACE2 activator, reduces spike-ACE2 interaction.

## Graphical abstract

**Figure:**
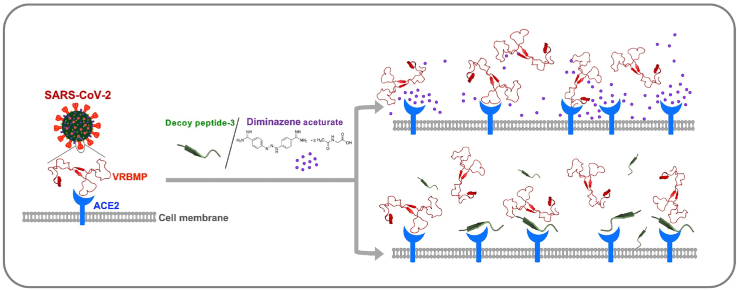

## 1 Introduction

At the end of 2019, a new type of virus causing a disease in the human respiratory system named severe acute respiratory syndrome coronavirus 2 (SARS-CoV-2) emerged in Wuhan, China. It was named after SARS-CoV, which was identified in 2003, and shared approximately 80 % nucleotide sequence similarity [[1], [2], [3]]. The virus quickly spread worldwide as the COVID-19 pandemic, reaching 664 million cases and 6.7 million deaths globally as of January 22, 2023. Both SARS-CoV-2 and SARS-CoV belong to the pathogenic beta coronavirus subgroup and are highly transmissible between humans [4,5]. SARS-CoV-2 is an enveloped spherical virus with a positive single-stranded RNA genome. The viral particle contains four structural proteins, the spike protein (S), the envelope protein (E), the membrane protein (M) and the nucleocapsid protein (N) [6].

SARS-CoV-2 first attaches to the surface of target cells, and entry into the cells is mediated by the binding of the viral spike glycoprotein to human angiotensin-converting enzyme 2 (ACE2) receptor. Then, protease enzymes, including furin, transmembrane protease serine 2 (TMPRSS2), and cathepsin-L, cleave the spike protein, resulting in the fusion of the virus particle and the host cell [[7], [8], [9]]. These mediating factors for viral entry such as ACE2 and TMPRSS2/Furin are widely enriched in epithelial cells of salivary glands and oral mucosa, which contribute to SARS-CoV-2 infection via respiratory and gastrointestinal routes [10,11]. Consistent with the virus entry mechanism, oral manifestations of COVID-19 include xerostomia, halitosis, dysgeusia or ageusia, burning sensation, stomatitis, aphthous-like ulcers, and severe sore throat that results in dysphagia [12,13]. Thus, the oral cavity may serve as one of the primary target sites for SARS-CoV-2 transmission, followed by systemic spread via the saliva to other deep tissues including the lungs and the digestive system [10,14,15]. Since the ACE2 receptor plays a crucial role in virus entry into the target cells, developing reagents that target ACE2 to inhibit virus binding to oral epithelial cells may effectively prevent COVID-19 infection.

Vaccines approved by the Food and Drug Administration (FDA), European Union, and other countries are currently the mainstream strategy to prevent the spread of COVID-19. However, due to the innate properties of RNA viruses to frequently mutate, many variants have evolved and will continue to evolve; this leads to breakthrough disease outbreaks [16]. Several studies have suggested that the development of blockers that target ACE2 receptors, which do not mutate, is a potential therapeutic strategy for preventing the spread of COVID-19. Suggested blockers include high-affinity neutralizing antibodies, peptide-based binders, small-molecule inhibitors targeting specific proteases, such as TMPRSS2 or furin, and soluble human ACE2, all of which can block the binding of spike protein's receptor-binding motif (RBM) to the ACE2 receptor [[17], [18], [19], [20], [21], [22], [23], [24], [25]]. Specifically, some ACE2 activators, such as xanthenone (XNT) or diminazene aceturate (DIZE), have been proposed as having therapeutic potential for COVID-19 as it has been suggested that these drugs may reduce pulmonary fibrosis during SARS-CoV-2 infection [26,27]. The therapeutic potential of DIZE for COVID-19 was further supported by *in silico* molecular docking studies, in which DIZE was positioned at the interface between the spike protein and ACE2 [28,29]. Notably, DIZE is an antiparasitic drug already approved by the FDA for the treatment of trypanosomiasis.

After reviewing the potential strategies for preventing the spread of the virus, we focused on the ACE2 which mediates initial virus attachment to human host cells. Our rationale is that targeting the machinery present in the host cells could circumvent the problems caused by high virus mutation rates and rapid emergence of new variants. In accordance with our focus on ACE2, structure-based drug design has been applied to various human ACE2 variants to accelerate design of therapeutics against SARS-CoV-2 [30]. Mechanistically, we aimed to use virus-derived peptides from the spike protein's RBM, term “decoy peptides”, that interrupt the binding of the virus. X-ray crystallography showed that the structures of spike-ACE2 complexes of SARS-CoV and SARS-CoV-2 are highly similar [8], both displaying hotspot residues–S19, Q24, F28, E35, A36, D38, Y41, Q42, Y83, E329, N330, and R357–of ACE2 in a cross-alignment analysis [31]. Such structural conservation suggests blocking the accessibility of ACE2 to spike protein could be a reasonable approach in developing therapeutic agents for SARS-CoV-2 prevention.

In this work, we examined the potential of using spike decoys to obstruct the accessibility of ACE2 receptors to SARS-CoV-2, thereby inhibiting viral attachment to host cells. To accomplish this objective, we constructed a cell-based platform to screen for compounds capable of interfering with viral attachment to host cells. The platform utilizes the oral epithelial cell line, NOK, and a fluorescently labeled spike RBM—virus RBM peptide (VRBMP)—as a virus binding reporter. Next, we designed seven short decoy peptides (8–14 amino acids in length), which were derived from the spike RBM, and assessed their efficacy in inhibiting virus binding to host cells. Lastly, in consideration that blocking ACE2 could represent a more general mechanism for preventing viral infection, the efficacy of diminazene aceturate (DIZE), an ACE2 activator, in impeding viral attachment was also evaluated.

## 2 Materials and methods

### 2.1 Cell culture and reagents

Six head and neck squamous cell lines were used in this study. CAL27 (ATCC Cat. No. CRL-2095) was obtained from the ATCC. SAS (JCRB No: JCRB0260) was procured from the Japanese Collection of Research Bioresources Cell Bank. OECM-1 [32] was originally provided by Dr. Ching-Liang Meng (National Defense Medical Center, Taiwan), and further contributed by Dr. Kuo-Wei Chang (Department of Dentistry, National Yang Ming Chiao Tung University, Taiwan). YMOC-1 [33] was generously gifted by Dr. Hsi-Feng Tu (Department of Dentistry, National Yang Ming Chiao Tung University, Taiwan). OC5 (human buccal carcinoma cells) and OC4 (human tongue carcinoma cells), initially sourced as primary cells, were transformed into cell lines through consecutive passages spanning more than 2 years. The normal oral keratinocytes cell line (NOK) [34] was originally provided by Dr. Shih-Yuan Peng and kindly shared by Dr. Kuo-Wei Chang. The 293 cell line (ATCC Cat. No. CRL-1573) and the non-small-cell lung carcinoma cell line H1299 (ATCC Cat. No. CRL-5803) was obtained from the ATCC.

The 293T, SAS, YMOC-1, and CAL27 cell lines were cultured in Dulbeccos Modified Eagle Medium (Gibco Cat. No.12100) supplemented with 10 % fetal calf serum (Gibco Cat. No. 10437028), 1 % l-Glutamine Solution (Biological Industries Cat. No.03-020-1B) and 1 % Penicillin-Amphotericin-Streptomycin (Corning 30-004-CI). The OECM-1 and H1299 cell lines were cultured in RPMI Medium 1640 (Gibco Cat. No.31800) supplemented with 10 % fetal calf serum, and 1 % Penicillin-Amphotericin-Streptomycin. The NOK cell line was cultured in Keratinocyte SFM (Gibco Cat. No.10724) supplemented with 10 % fetal calf serum, 0.005 μg/ml EGF, 50 μg/ml BPE, and 1 % Penicillin-Amphotericin-Streptomycin. The OC4 cell line was cultured in Dulbecco's Modified Eagle Medium/Nutrient Mixture F-12 (Gibco Cat. No.12500) supplemented with 10 % fetal calf serum and 1 % Penicillin-Amphotericin-Streptomycin. The OC5 cell line was cultured in 45 % Dulbecco's Modified Eagle Medium/Nutrient Mixture F-12, 45 % Dulbecco's Modified Eagle Medium supplemented with 10 % fetal calf serum and 1 % Penicillin-Amphotericin-Streptomycin. All of the above cell lines were cultured at 37 °C in an atmosphere containing 5 % CO2; in all cases passages 3 to 7 were used for this research.

### 2.2 Western blotting

Cells were washed twice with cold ice-cold phosphate-buffered saline (PBS) containing the protease inhibitor phenylmethylsulfonyl fluoride (PMSF) at 10 μM/ml, Cells were harvested and disrupted by adding an appropriate volume of lysis buffer (50 mM Tris pH 7.5, 100 mM sodium chloride, 5 % sodium deoxycholate, 10 % Protease Inhibitor Cocktail (EDTA-Free), 25 μM phenylmethylsulfonyl ﬂuoride, and 0.2 μM sodium orthovanadate). After vortexing for 30 s, the cell lysates were frozen overnight at −80 °C, and this was followed by centrifugation at 13,500 × rpm for 30 min at 4 °C. Each supernatant was considered a cell extract. Protein concentrations were measured using a Bradford Protein Assay kit (Bio-Rad Cat. No.5000001). The proteins in each cell extract were separated by 8 % SDS-PAGE. After separation, the proteins were transferred to a nitrocellulose (NC) membrane using the Bio-Rad Mini Trans-Blot system. Thereafter a standard protocol was followed. First, each membrane was treated individually with one of two diluted primary antibodies, namely: anti-ace2 Ab (Sigma-Aldrich Cat. No. SAB3500346; 1: 1000), and anti-actin Ab (Merck Millipore Cat. No. MAB1501, 1:2000) in 5 % nonfat dry milk at 4 °C with shaking for 16 h, Next, the nitrocellulose membrane was washed twice with PBS containing 1 % Tween-20. This was followed by incubation with the appropriate secondary antibodies, namely anti-mouse IgG (Merck Millipore Cat. No. #AP124P, 1:1000) or anti-rabbit IgG (Merck Millipore Cat. No. #AP132P, 1:1000).

### 2.3 mRNA purification and reverse transcription‐quantitative polymerase chain reaction (RT-PCR)

Total RNA was extracted using TRIzol Reagent (Roche Life Science Cat. No. 11667165001), The cells after harvesting were disrupted by adding 0.75 ml of TRIzol per 1 × 10^5^ to 10^7^ cells. After homogenization of the disrupted cells, 0.15 ml of 1-Bromo-3-chloropropane (Sigma-Aldrich Cat. No. B9673) per 0.75 ml of TRIzol was added and the mixture shaken gently 10 times, which was followed by incubation for 10 min at room temperature. Next, the sample was centrifuged at 13,500 × rpm for 15 min at 4 °C and then the aqueous phase containing the RNA was transferred to a new tube.To this tube, 0.375 ml of isopropanol (JT Baker Cat. No. 9084-03) was added and this was followed by gentle shaking 10 times, then incubation for 10 min at room temperature. At this point, each sample was centrifuged at 13,500 × rpm for 30 min at 4 °C. The supernatant was discarded and 0.6 ml of 75 % ethanol added to the pellet. The tube was centifuged again, this time at 13,500 × rpm for 5 min at 4 °C. Finally, the pellet was disolved in Tris-EDTA buffer and the RNA concentration in this supernatent determined by the Nanodrop method (all absorbance A260/280 ratios were between 1.8 and 2.0 and all A260/230 ratios were between 2.0 and 2.2). The total RNA in solution was treated with DNAse-I from a DNase Assay Kit (Promega Cat. No. N251B, M610A) according to the manufacturer's instructions and then reverse transcribed into cDNA using a MMLV High Performance Reverse Transcriptase Kit (Lucigen Cat. No. RT80125K) according to the manufacturer's instructions. qPCR was performed using a 20 μl volume containing 3.4 μl cDNA, 10 μl TaqMan™ Universal Master Mix II, no UNG (Applied Biosystems, Cat. No.4440049), 1 μl TaqMan probe (Thermofisher Cat.No. Hs01085333_m1; Hs00266705_g1), and 5.6 μl Nuclease- Free Water (Invitrogen Cat.No. AM9938). The thermocycling conditions were as follows: 50 °C for 2 min, 95 °C for 10 min then 40 cycles of 95 °C for 15 min, 60 °C for 1 min. The relative fold change of each target gene was analyzed using the 2-ΔΔCt method. GAPDH was used as the internal control.

### 2.4 Peptide design and synthesis

ACE2 decoy peptides originate from specific fragments within the spike receptor binding motif (RBM) of SARS-CoV-2. These peptides range in length from approximately 8 to 14 amino acids, and those peptides encompass direct binding between the RBM and ACE2. Two of these peptides, specifically VRBMP-1 (Virus Receptor Binding Motif Peptide 1) and VRBMP-2 (Virus Receptor Binding Motif Peptide 2), were utilized to emulate viral interactions. These peptides encompass the sequence spanning from amino acid positions 438 to 506 within the Spike RBM and FITC (Fluorescein isothiocyanate) was attached to the C-terminus of these peptides to facilitate visualization and analysis. Notably, VRBMP-2 retains a crucial dual cysteine bond structure (Cys480 and Cys488), which potentially contributes to upholding its structural stability attributes. Instead, Neg-1 (Negative Control Peptide 1) and Neg-2 (Negative Control Peptide 2) were designed as control peptides. These peptides encompass the sequence spanning from amino acid positions 477 to 494 within the RBM. In these sequences, the amino acids have been randomly rearranged. Additionally, FITC was conjugated to the C-terminus of these peptides. Peptide*s* were manufactured by BIOTOOLS and Allbio Life Co., Ltd, purified to >98 % purity. Each peptide consisted of a lyophilized freeze-dried powder and these were dissolved in either DMSO or Acetonitrile and then kept at −20 °C until use.

### 2.5 Confocal microscope analysis in ACE2 decoy peptide-treated NOK cells

A total of 2 × 10^4^ cells in 500 μl culture medium was seeded into 24 well cell culture plates containing coverslips and incubated overnight in a 37 °C in a 5 % CO_2_ incubator. Next the cells are washed once with 0.35 ml KSFM and replenished with 0.35 ml KSFM containing different concentrations (25 μg/ml, 100 μg/ml, 250 μg/ml) of a decoy peptide for a specific time. In the following step, the medium surrounding the cells was replaced with 0.35 ml KSFM containing 50 μg/ml or 100 μg/ml of one of the following peptides: VRBMP-1, VRBMP-2, Neg-1, or Neg-2 for 24 h. Finally, the cells underwent immunofluorescence microscopy.

### 2.6 ACE2-neutralizing antibodies

A total of 2 × 10^4^ cells in 500 μl of culture medium was seeded into 24 well cell culture plates containing coverslips and incubated overnight in a 37 °C, 5 % CO_2_ atmosphere incubator. Next the cells are washed once with 0.35 ml KSFM and each well replenished with 0.35 ml KSFM containing different concentrations (2 μg/ml or 20 μg/ml) of ACE2 antibodies (Sigma-Aldrich Cat. No. SAB3500346). After 30 min, the cell medium was replaced with 0.35 ml KSFM containing 50 μg/ml of either VRBMP-1 or Neg-1 peptide for 24 h. Finally the cells underwent immunofluorescence microscopy.

### 2.7 Immunofluorescence

Each sample was washed twice with PBS in the 24 well cell culture plates and this was followed by fixing the cells by adding 0.35 ml of 4 % paraformaldehyde for 15 min at room temperature. After removal of the fixative, the cells were permeabilized by adding 0.35 ml of PBS containing 0.1 % Triton X-100 for 10 min at room temperature. The Triton X-100 was then removed and coverslips with the cells attached were blocked by adding 50 μl of PBS containing 10 % normal goat serum per coverslip; the coverslips were then incubated for 1 h at room temperature. Next the coverslips were incubated in primary antibody (Sigma-Aldrich Cat. No. SAB3500346; 1: 200, R&D Cat. No.AF933; 1:200) in PBS overnight at 4 °C. After this, the samples were washed two times with PBS, which was followed by incubation with appropriate secondary antibodies (Jackson Immuno Cat.111-005-003; 1:200, Abcam Cat.ab150131; 1:200) and DAPI (Sigma-Aldrich Cat.D9542; 1:1000) in PBS for 1hr at room temperature. Each coverslip was then mounted using a drop of mounting medium (Sigma-Aldrich Cat.F4680). Images were captured using a confocal laser scanning microscope (Olympus FV10i).

### 2.8 Protein structural analysis

The crystal structure of the SARS-CoV-2 Spike (S) protein-ACE2 complex (PDB: 7KNB) was downloaded from the RCSB-PDB website. Residues of ACE2 interacting with residues of the spike RBD were then identified by using distance measurements in the PyMOL software. All 3D structures were generated with PyMOL.

### 2.9 Decoy peptides 3D structure and primary structure prediction

Decoy peptides were created and consisted of between 8 and 14 amino acids; these had similar 3D structures as predicted by PEP-FOLD (https://bioserv.rpbs.univ-paris-diderot.fr/services/PEP-FOLD/) and these were then modified with PyMOL.

### 2.10 ACE2-SARS-CoV-2 RBD binding inhibition ELISA

Microtiter wells of ELISA plates were coated with Recombinant Human ACE-2 Fc Chimera (R&D Systems Cat. No.10544-ZN) at 20 ng/100 μl per well. Coating was performed overnight at 4 °C to immobilize ACE-2 Fc Chimera. Plates were subsequently blocked to prevent non-specific binding. After blocking, plates were pre-treated with either DMSO (control) or various concentrations of DP-3 or DIZE for 1 h at room temperature. Following pre-treatment, each well received 50 ng of recombinant SARS-CoV-2 Spike RBD His Biotin Protein, CF (R&D Systems Cat. No.BT10500) in 100 μl. ELISA analyses were conducted by utilizing a commercially available DuoSet ELISA Ancillary Reagent Kit 2 (R&D Systems Cat. No.DY008B) and Streptavidin-HRP (R&D Systems Cat. No.DY998) according to the manufacturer's instructions. Absorbance measurements were obtained at 450 nm using a multimode microplate reader (Tecan, Infinite 200pro).

### 2.11 Plasmids and cell subpopulations

The coding sequences of ACE2 were amplified from OC4 cell line cDNA through PCR. The amplification process utilized the forward primer: 5′-GGGATCAGAGATCGGAAGAAGAAA-3′ and the reverse primer: 5′-AGGAGGTCTGAACATCATCAGTG-3’. These amplified sequences were subsequently cloned into pcDNA3.1(−) vectors (Addgene Cat. No. V79520). For this cloning, we employed forward and reverse primers that incorporated *Xba*I and *Xho*I restriction sites, respectively. The forward primer used was: 5′-GGCTCTAGA ATGTCAAGCTCTTCCTGG-3′, and the reverse primer was: 5′-CCGCTCGAGCTAAAAGGAGGTCTGAACA-3’. Validation of the reporter construct was conducted through restriction digestion and DNA sequencing, followed by alignment against publicly available sequence data.

As part of the control experiment, an empty vector devoid of the ACE2 gene (pCDNA3.1(−)) was employed. Transfection of 293 cells was executed using the pcDNA3.1(−)-hACE2 clones. Specifically, 5 × 10^5^ cells were seeded in 6-well plates and transfected with either the expression vector or the empty vector (1 μg) using TransFectin Lipid Reagent (BioRad Cat No. 1703351). Transfection was carried out for 24 h, adhering to the manufacturer's instructions.

### 2.12 Statistical analysis and fluorescence quantification

Statistical analysis was carried out using GraphPad Prism (version 9). Data in figures were presented as mean ± SEM and unpaired *t*-tests with equal variance were then used to assess statistical significances. *P* values less than 0.05 were considered significant. Fluorescence was quantified linearly on each individual experiment; thus, the arbitrary unit (a.u.) of fluorescence intensity is not comparable across different figures.

### 2.13 AI-assisted writing

During the preparation of the manuscript, ChatGPT (GPT-4 May 24 Version) was used for grammatical correction and semantic recommendation. For each paragraph, we created a first draft that was completely based on the facts, findings, and reasonings of our own. Next, we let ChatGPT make suggestions by specifying the prompt with “in the style of scientific writing” and “without changing its meaning”. In most cases ChatGPT produced writings that were overly verbose but with higher semantic accuracy. Lastly, based on ChatGPT's writing, we made grammatical corrections and semantic improvements on our original draft. This very paragraph describing AI-assisted writing has not been subjected to any AI technology.

## 3 Results

### 3.1 Angiotensin-converting enzyme 2 (ACE2) receptor is highly expressed in oral epithelial cells

**Fig. 1:**
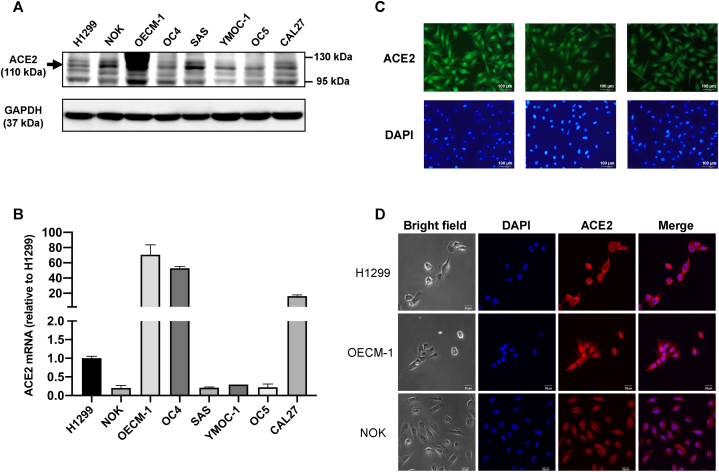
Expression of angiotensin-converting enzyme 2 (ACE2) on oral epithelial cells. (A) Western blot analysis of ACE2 protein in various oral epithelial cells: NOK, OECM-1, OC4, SAS, YMOC-1, OC5, and CAL27. The non-small-cell lung cancer cell line, H1299, was used as the positive control, with GAPDH as the loading control. (B) Real-time qRT-PCR analysis of ACE2 mRNA levels in various cell lines, presented as fold change relative to the H1299 cell line. Error bars represent SEM (n = 2). (C) Representative fluorescence microscopic images showing immunostaining of ACE2 (green) and nuclei staining with DAPI (blue) in NOK cells. Scale bars indicate 200 μm. (D) Representative confocal images of ACE2 (red) with nuclei stained by DAPI (blue) in H1299, OECM-1, and NOK cells. Scale bars indicate 20 μm. (For interpretation of the references to colour in this figure legend, the reader is referred to the Web version of this article.)

One of the critical receptors for SARS-CoV-2 viral entry is ACE2. To investigate endogenous ACE2 expression in various oral cell lines, we analyzed several oral epithelial cell lines, including cancer cell lines such as OECM-1, OC4, SAS, YMOC-1, OC5, and CAL27, as well as an immortalized normal oral epithelial cell line, NOK. We also used H1299, a lung carcinoma cell line, as the positive control (Fig. 1A and B). The specificity of anti-ACE2 antibody was validated by comparing empty vector control and over-expression in 293 cells (Fig. S1A). Notably, NOK, OECM-1, OC4, SAS, YMOC-1, and CAL27 exhibited higher levels of ACE2 expression than H1299 at the protein level (Fig. 1A), and all of the oral epithelial cells showed some level of ACE2 mRNA expression (Fig. 1B). Specifically, OECM-1, OC4, and CAL27 displayed exceptionally high levels of ACE2 mRNA—approximately 20 to 60 times higher than that of H1299 (Fig. 1B). Furthermore, by utilizing immunofluorescence (Fig. 1C) and confocal microscopy (Fig. 1D), we observed that ACE2 was predominantly localized on the cellular membranes, with some presence in the cytoplasm (Fig. 1D and Fig. S1B). As opposed to cell lines derived from cancer tissues, NOK was directly immortalized from normal keratinocytes, which better mimics normal physiologic states. Therefore, we chose NOK as the primary host cell line for this study.

### 3.2 Key amino acid residues in the SARS-CoV-2 RBD–ACE2 complex

**Fig. 2:**
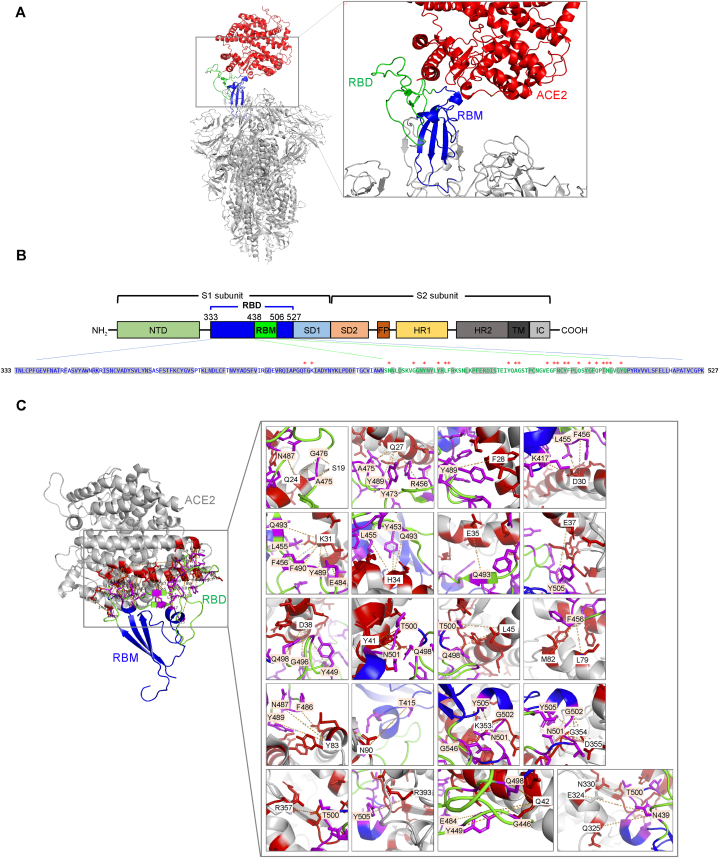
Structural analysis of the binding interface between SARS-CoV-2 spike RBD and ACE2. (A) Overview of the SARS-CoV-2 spike protein (gray) bound to human ACE2 (red) (PDB ID: 7KNB). The receptor binding domain (RBD) and receptor binding motif (RBM) of the spike protein were shown in blue and green, respectively. (B) Domain schematics of the SARS-CoV-2 spike protein adopted from Lan et al. [8]. FP, fusion peptide; HR1, heptad repeat 1; HR2, heptad repeat 2; IC, intracellular domain; SD1, N-terminal subdomain 1; SD2, N-terminal subdomain 2; TM, transmembrane region. Bottom panel: residues 333 to 537 of spike protein, i.e., the RBD. The RBM sequence was shown in green fonts. Residues conserved across SARS-CoV-1 and SARS-CoV-2 were highlighted in gray shades. Red asterisks mark residues in direct contact with the ACE2 receptor. (C) Detailed structural analysis of the binding interface between SARS-CoV-2 spike protein and ACE2. The ACE2 receptor, RBD, and RBM of the spike protein were depicted in gray, blue, and green, respectively. In the right insets, specific residues involved in direct interaction were shown in magenta and red for the spike protein and ACE2, respectively. Residue labels were shown with light yellow and white shades for the spike protein and ACE2, respectively. (For interpretation of the references to colour in this figure legend, the reader is referred to the Web version of this article.)

In order to design peptides with the potential to inhibit the binding of the SARS-CoV-2 spike protein, it is crucial to understand the structural interactions between the ACE2 and the spike protein. The crystal structure of the SARS-CoV-2 spike-ACE2 complex was obtained from the Protein Data Bank (PDB) database (entry: 7KNB) [35] and subsequently visualized and analyzed using PyMOL software (Fig. 2). The spike protein (1273 amino acids) is composed of three identical subunits, each containing a receptor-binding domain (RBD) capable of interacting with ACE2 (Fig. 2A). The RBD spans between residues 330 and 527, and contains a receptor-binding motif (RBM), which directly interacts with ACE2 (Fig. 2A and B). The RBD exhibits extensive glycosylation, a characteristic shared by the entire spike protein. SARS-CoV-2 employs its RBD to target a specific region on the human ACE2 receptor, encompassing residues S19 to R393 [8] (Fig. 2A and B), including several hotspot residues: S19, Q24, F28, E35, A36, D38, Y41, Q42, Y83, E329, N330, and R357 [31]. We thoroughly reviewed the literature and identified key residues at the RBD-ACE2 interface (Fig. 2C and Table 1). In the crystal structure, these key residues were localized within the RBM, in a way that various segments of amino acid sequences were in close proximity with ACE2 (Fig. 2C). Using the structural information of the RBM sequence, we designed 7 decoy peptides of 8–14 amino acids in length, such that these peptides maximally encompass the key interaction residues (Fig. 4A).

**Table 1:** Key residues within the human ACE2 receptor and virus spike RBD that interact with each other.

hACE2	SARS-CoV2-RBD	Support SARS-CoV infection	References
S19	A475, G476	–	[[36], [37], [38], [39]]
Q24	A475, G476, N487	–	[8,[36], [37], [38], [39]]
D30	K417, L455, F456	–	[8,36,38,39]
T27	F456, Y473, A475, Y489	–	[36,38]
F28	Y489	–	[36,38]
K31	L455, F456, E484, Y489, Q493, F490	Yes	[[36], [37], [38], [39], [40]]
H34	Y453, L455, Q493	–	[8,[36], [37], [38], [39]]
E35	Q493	Yes	[[36], [37], [38], [39], [40]]
E37	T505	–	[36,38]
D38	Y449, G496, Q498	Yes	[31,[37], [38], [39], [40]]
Y41	Q498, T500, N501	Yes	[31,[36], [37], [38], [39],^41^]
Q42	G446, Y449, E484, Q498		[31,[36], [37], [38], [39],^41^]
L45	Q498, T500	–	[[36], [37], [38],^41^]
L79	F486		[8,36,38,41]
M82	F486	Yes	[8,36,38,40,41]
Y83	F486, N487, Y489	–	[8,36,38,39,41]
N90	T415	–	[38,40,41]
Q325	N439	–	[38,41]
E329	N439	–	[31,41]
N330	T500	–	[[36], [37], [38],^41^]
K353	G496, N501, G502, Y505	Yes	[[36], [37], [38], [39], [40]]
G354	Y502, Y505	–	[36,38]
D355	N501, G502	–	[[36], [37], [38]]
R357	T500	–	[36,38]
R393	Y505	–	[36,38]

### 3.3 Synthetic virus-mimicking peptides, VRBMPs, displayed potent binding capacity to ACE2 on oral epithelial cells

**Fig. 3:**
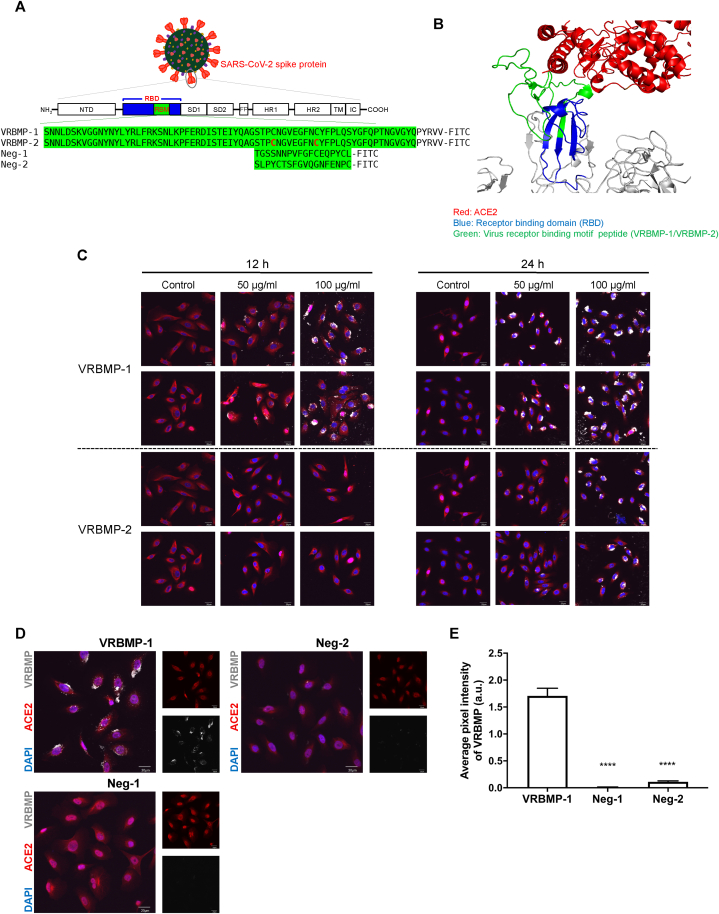
VRBMP exhibits strong binding capacity to ACE2 on oral keratinocytes. (A) Schematics of the sequences of the synthesized peptides VRBMP-1/VRBMP-2 and two negative-control peptides, Neg-1 and Neg-2. All peptides were covalently conjugated with a fluorescein isothiocyanate (FITC) label at C-termini. (B) Structure of the binding interface between spike protein and ACE2 showing VRBMP sequences in green. (C) Confocal fluorescence microscopic images of NOK cells incubated in media containing 50 or 100 μg/ml of VRBMP-1 or VRBMP-2 for 12 and 24 h. ACE2, red; DAPI, blue; VRBMP-1/VRBMP-2, light gray. Scale bars indicate 20 μm. (D) Confocal images of NOK cells after treatment with 50 μg/ml of VRBMP-1, Neg-1, or Neg-2 for 24 h. ACE2, DAPI, and VRBMP were shown in red, blue, and light gray, respectively. Scale bars indicate 20 μm. (E) Quantification of average VRBMP signal intensity in 15 fields of confocal images. Data were presented as mean ± SEM (n = 15) with statistical analysis using unpaired *t*-tests with equal variance (****p < 0.0001). (For interpretation of the references to colour in this figure legend, the reader is referred to the Web version of this article.)

Research on hazardous pathogens like SARS-CoV-2 is often restricted to high-level biosafety laboratories due to safety concerns. Nevertheless, virus mimicking techniques can provide a controlled and safe environment for conducting research. To mimic the SARS-CoV-2 spike protein, we further analyzed the crystal structure and found that the RBM contained most of the residues that directly bind to the ACE2 receptor, including residues 438 to 506 of the spike protein. Thus, we synthesized two long peptides, designated as VRBMP-1 and VRBMP-2 (virus receptor-binding motif peptide-1 and peptide-2), which were labeled with fluorescein isothiocyanate (FITC) at their C-termini. There are four disulfide bridges within the RBD of SARS-CoV-2. Among these, the one formed between Cys480 and Cys488 appears to be critical for virus entry into host cells [42,43]. VRBMP-2 contains this specific oxidized disulfide bridge, while VRBMP-1 contains two reduced cysteines without disulfide linkage (Fig. 3A and B).

**Fig. 4:**
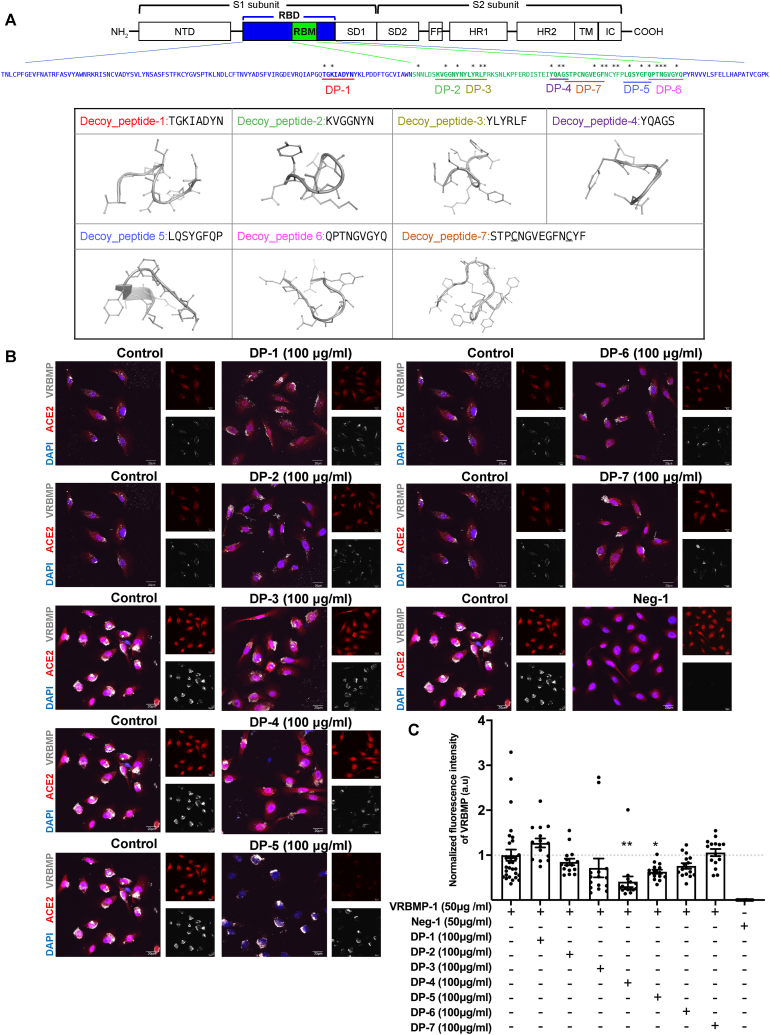
Candidate decoy peptides that inhibit VRBMP-1 binding to ACE2. (A) Top panel: sequences of decoy peptides were underlined within the RBD sequence. Red asterisks indicate residues in direct contact with ACE2. Bottom panel: structures of decoy peptides predicted by the software PEP-FOLD [44]. For decoy peptide-7, two cysteine residues forming a disulfide linkage were underlined. (B) Confocal fluorescence microscopic images of NOK cells incubated in media containing decoy peptides at 100 μg/ml for 1 h and then shifted to 50 μg/ml of VRBMP-1 or Neg-1 for another 24 h. ACE2, DAPI, and VRBMP-1 are shown in red, blue, and light gray, respectively. Scale bar indicates 20 μm. (C) Quantification of average VRBMP signals in NOK cells. Fluorescence intensities of decoy peptides were normalized to the VRBMP-1 mock control. Data are presented as mean ± SEM (n = 15) with statistical analysis using unpaired *t*-tests with equal variance (*p < 0.05, **p < 0.01). (For interpretation of the references to colour in this figure legend, the reader is referred to the Web version of this article.)

We then tested whether VRBMP-1 and VRBMP-2 can bind to oral epithelial cells. NOKs were cultivated in the presence of VRBMP-1 or VRBMP-2 at 50 and 100 μg/ml for 12 or 24 h. Confocal microscopy showed colocalization of ACE2 and VRBMPs, in a dose-dependent and time-dependent manner (Fig. 3C). Conversely, two negative-control peptides, Neg-1 and Neg-2, were created by randomly shuffling the residues between 450 and 477 of the spike protein, which are critical to ACE2 binding. Neg-1 and Neg-2 did not show any discernible binding to NOK cells, indicating that only peptides with a specific amino acid sequence from the RBM of spike protein can bind to ACE2 (Fig. 3D and E). Interestingly, the disulfide-containing VRBMP-2 showed a lower fluorescence intensity than VRBMP-1, suggesting that the disulfide bond may reduce the affinity of RBM to ACE2, despite its positive effect on viral entry into the host cell (Fig. 3C).

To validate our virus mimicking platform that VRBMPs specifically interact with ACE2 on oral epithelial cells, we used an anti-ACE2 antibody to interrupt VRBMP-ACE2 interaction. NOK cells were immersed in 2 or 20 μg/ml of the anti-ACE2 antibody in a culture medium for 30 min at 37 °C, before 50 μg/ml of VRBMP-1 was added for another 24 h. A nonspecific IgG served as the control. The results showed that anti-ACE2 antibody reduced the binding between VRBMP and ACE2 by 15 fold (Fig. S2). Taken together, we constructed a cell-based platform in which the virus-mimicking VRBMPs specifically binds to ACE2, serving as a proxy for virus attachment. This platform was used to evaluate the efficacy of small decoy peptides and small-molecule inhibitors in subsequent experiments.

### 3.4 Decoy peptides effectively inhibit VRBMP-1 binding to oral keratinocytes

The construction of the VRBMP platform allowed us to efficiently test the efficacy of candidate decoy peptides in inhibiting virus attachment. We used PEP-FOLD [44] to predict the solution structure of the decoy peptides, and found that most of them formed loop structures with internal hydrogen bonds between side chains (Fig. 4A). Decoy peptides were applied to NOK cells at a concentration of 100 μg/ml for 1 h. Next, 50 μg/ml of VRBMP-1 or Neg-1 was added to the culture medium and incubated for another 24 h. The immunofluorescent intensity data demonstrated that most decoy peptides, specifically DP-2, 3, 4, 5, and 6, effectively inhibited VRBMP-1 binding to NOK cells (Fig. 4B and C). The inhibitory effect of decoy peptide, specifically DP-3, on ACE2-RBD interaction was further confirmed through an independent ELISA assay similar to those used in previous studies [[45], [46], [47]] (Figs. S3A–C). Intriguingly, we found that ACE2 expression was decreased in the presence of DP-5, although the underlying mechanism remains unknown. Collectively, these findings indicate that decoy peptides competitively inhibit the binding of VRBMP-1 to ACE2 receptors, possibly obstructing virus entry into host cells.

### 3.5 Combinatorial low concentrations of decoy peptides effectively block VRBMP-1 binding

**Fig. 5:**
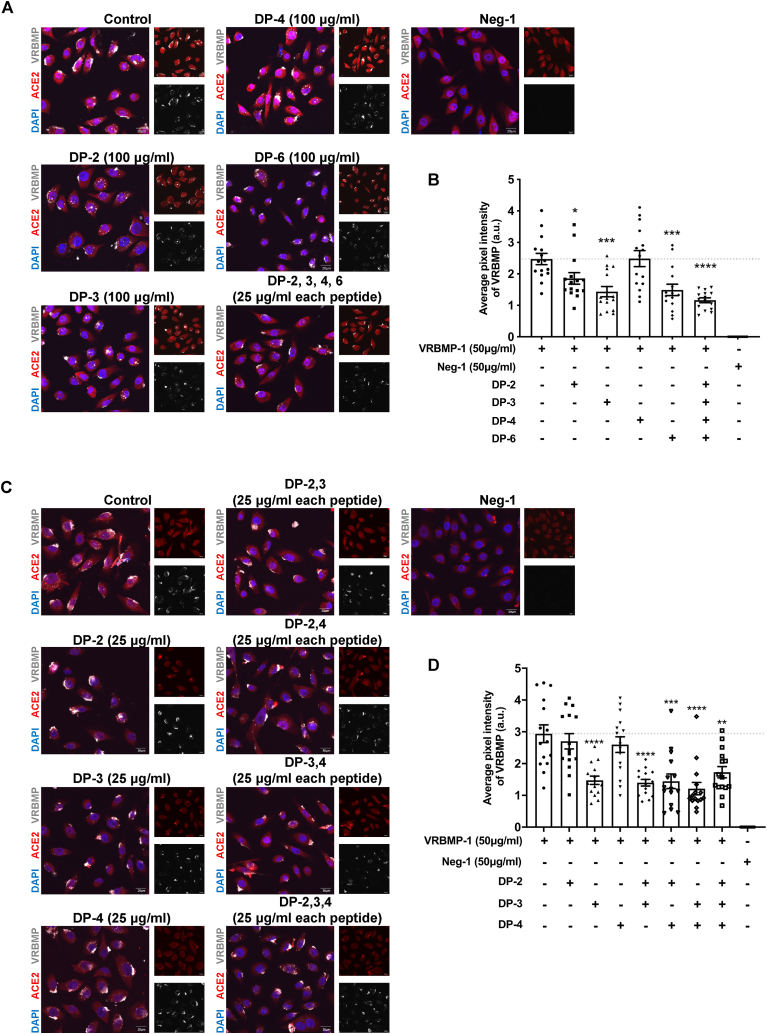
Optimizing combinations of decoy peptides to enhance competitive inhibition of VRBMP binding. (A, B) Confocal fluorescence microscopic images showing NOK cells incubated in media containing decoy peptides at 100 μg/ml for 1 h and then shifted to 50 μg/ml of VRBMP-1 or Neg-1 for another 24 h. For the combination of decoy peptides-2, 3, 4, and 6, 25 μg/ml of each peptide were combined. ACE2, DAPI, and VRBMP-1 are shown in red, blue, and light gray, respectively. Scale bars indicate 20 μm. (C, D) Confocal fluorescence microscopic images showing NOK cells incubated in media containing single peptide or combinations two or three peptides (each at 25 μg/ml) for 1 h and then shifted to 50 μg/ml of VRBMP-1 or Neg-1 for another 24 h. ACE2, DAPI, and VRBMP-1 are shown in red, blue, and light gray, respectively. Scale bars indicate 20 μm. (B, D) Quantification of average VRBMP signals. Error bars indicate SEM (n = 15). *P* values (***p < 0.001, ****p < 0.0001) were determined by unpaired *t*-test with equal variance. (For interpretation of the references to colour in this figure legend, the reader is referred to the Web version of this article.)

Given that the decoy peptides were derived from different segments of the spike protein's RBM, we postulated that combinations of these peptides could lead to synergistic effects in terms of inhibition. To investigate this hypothesis, we conducted a combinatorial analysis that involved decoy peptides DP-2, 3, 4, and 6, as these peptides exhibited a higher degree of inhibitory efficacy (Fig. 5). We set a lower concentration (25 μg/ml) for each peptide in the combination. Compared to individual decoy peptides at 100 μg/ml, the combination of DP-2, 3, 4, and 6 with each peptide at 25 μg/ml displayed significantly stronger inhibition (Fig. 5A and B). Subsequently, we asked whether it was possible to achieve a similar boost in efficacy using combinations consisting of fewer decoy peptides, specifically two or three peptides. Results showed that none of the pair and triple combinations performed better than DP-3 alone (Fig. 5C and D). In conclusion, our results indicate that the synergistic inhibition of the binding between the VRBMP-1 and ACE2 can be successfully achieved through a combination of four decoy peptides, with each peptide present at a lower concentration than when used individually.

### 3.6 Intensive short-term treatment with higher doses of decoy peptide strongly inhibits VRBMP-1 binding

**Fig. 6:**
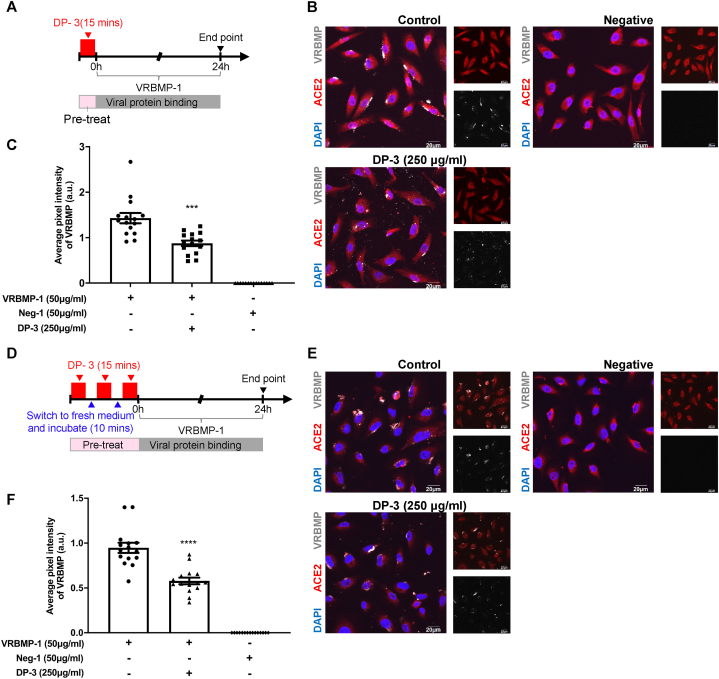
Short treatment period of decoy peptide was sufficient to inhibit VRBMP-1 binding to ACE2. (A) Schematics of the experimental timeline for the 15-min DP-3 treatment. (B) Confocal fluorescence microscopic images showing NOK cells incubated in media containing DP-3 at 250 μg/ml for 15 min and then shifted to 50 μg/ml of VRBMP-1 or Neg-1 for another 24 h. ACE2, DAPI, and VRBMP-1 are shown in red, blue, and light gray, respectively. Scale bars indicate 20 μm. (C) Quantification of average VRBMP signals in (B) using 15 image fields. Error bars indicate SEM (n = 15). (D) Schematics of the experimental timeline for three consecutive 15-min DP-3 treatment sessions. (E) Confocal fluorescence microscopic images showing NOK cells incubated in media containing DP-3 at 250 μg/ml for three consecutive 15 min sessions, with 10-min fresh medium wash between each treatment, and then shifted to 50 μg/ml of VRBMP-1 or Neg-1 for another 24 h. ACE2, DAPI, and VRBMP-1 are shown in red, blue, and light gray, respectively. Scale bars indicate 20 μm. (F) Quantification of average VRBMP signals in (E) using 15 image fields. Error bars indicate SEM (n = 15). All *P* values (***p < 0.001, ****p < 0.0001) were determined by unpaired *t*-test with equal variance. (For interpretation of the references to colour in this figure legend, the reader is referred to the Web version of this article.)

Next, we explored the feasibility of reducing the treatment duration of decoy peptides to achieve rapid blocking effects, since administration duration could be critical in developing therapeutic strategies. To achieve this, we elevated the concentration of DP-3 to 250 μg/ml and decreased the treatment duration to 15 min (Fig. 6A). Our results demonstrated that one session of 15-min treatment effectively impeded the binding between VRBMP-1 and ACE2 (p < 0.001) (Fig. 6B and C). Moreover, when NOK cells were exposed to three sequential 15-min treatment sessions, a stronger inhibition of VRBMP-1-ACE2 interaction was observed (p < 0.0001) (Fig. 6D–F). In summary, our findings suggest that a shortened treatment duration coupled with increased decoy peptide concentration can efficiently disrupt the VRBMP-1-ACE2 interaction, offering the potential strategies for therapeutics development.

### 3.7 Diminazene aceturate (DIZE), an ACE2 activator, effectively inhibits VRBMP-1 binding to ACE2 in oral keratinocytes

**Fig. 7:**
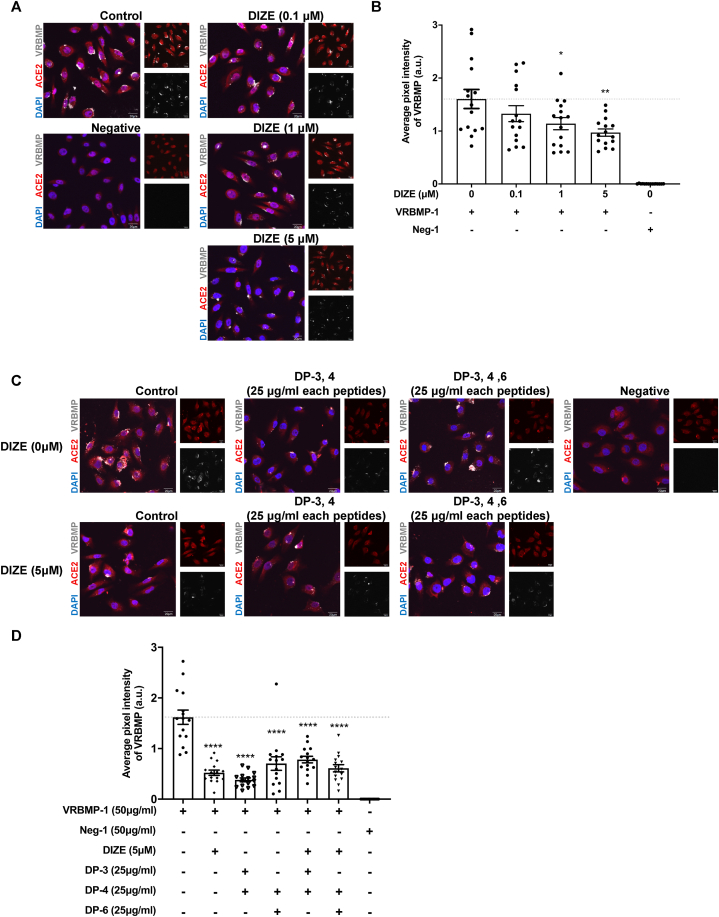
Diminazene aceturate (DIZE) inhibits VRBMP binding to the ACE2 receptor. (A) Confocal fluorescence microscopic images showing NOK cells cultured in media containing various concentrations of DIZE (0, 0.1, 1, and 5 μM) for 1 h and then shifted to 50 μg/ml of VRBMP-1 or Neg-1 for another 24 h. ACE2, DAPI, and VRBMP-1 were shown in red, blue, and light gray, respectively. Scale bars indicate 20 μm. (B) Quantification of average VRBMP signals in NOK cells. Data are presented as mean ± SEM (n = 15) with statistical analysis using unpaired t-tests with equal variance (*p < 0.05, **p < 0.01). (C) Confocal fluorescence microscopic images showing NOK cells incubated with different combinations of decoy peptides (25 μg/ml each peptide) and 5 μM of DIZE for 1 h and then shifted to 50 μg/ml of VRBMP-1 or Neg-1 for another 24 h. Scale bars indicate 200 μm. (D) Quantitative analysis VRBMP signals in (C) with error bars indicating SEM (n = 15). *P* values (****p < 0.0001) were determined by unpaired *t*-test with equal variance. (For interpretation of the references to colour in this figure legend, the reader is referred to the Web version of this article.)

The effectiveness of synthetic decoy peptides led us to ask whether blocking ACE2's availability could be generalized to prevent virus attachment. We conducted a literature search for chemical agents that could potentially achieve this purpose [43,48]. One such compound is diminazene aceturate (DIZE), an ACE2 activator, which has been used clinically to treat trypanosomiasis or arrhythmia. Given that DIZE was shown to be positioned at the interface between RBD and ACE2, in close proximity to interacting residues including D33, H34, E37, D38, K353 [28], we hypothesized that DIZE could reduce the binding of ACE2 with spike protein. By using the same approach, we treated NOK cells with or without DIZE at various concentrations (0.1, 1, and 5 μM) for 1 h, followed by incubation with 50 μg/ml of VRBMP-1 or Neg-1 for another 24 h. The DIZE-treated cells showed significantly reduced binding of VRBMP-1 at all three concentrations in a dose-dependent manner (Fig. 7A and B). The inhibitory effect of DIZE was further confirmed through independent ELISA assays (Fig. S3D). Then, we asked whether DIZE had a synergistic effect with decoy peptides on the suppression of VRBMP-1 binding to ACE2 receptors. NOK cells were treated with 25 μg/ml of each peptide with or without 5 μM of DIZE for 1 h, and then shifted to 50 μg/ml of VRBMP-1 or Neg-1 for 24 h. Consistent with previous findings, both decoy peptides and DIZE significantly reduced the binding of VRBMP-1 with nearly the same potency. It is noted that the molarities of decoy peptides and DIZE are different and hence not directly comparable. Specifically, 25 μg/ml of DP-3 is equivalent to 28.5 μM, which is approximately five to six times higher than the molarity of DIZE. Intriguingly, combing DIZE and decoy peptides resulted in less inhibition, compared to those of each alone (Fig. 7C and D). This implies that target sites of the decoy peptides and DIZE on the ACE2 receptors may overlap, resulting in a diminishing effect when both were used simultaneously.

In conclusion, this study demonstrated that spike decoys block ACE2 accessibility on oral epithelial cells. Spike decoy peptides were tested on a newly developed cell-based platform which centers on spike-ACE2 interaction. We also showed that the ACE2 activator, DIZE, exerts similar effects as decoy peptides, suggesting that blocking ACE2 accessibility could be a robust strategy in preventing SARS-CoV-2 infection.

## 4 Discussion

The battle against COVID-19 requires not only vaccination but also the development of novel therapeutics, thereby broadening the toolkits available for comprehensive pandemic management. A variety of drugs, e.g. Actemra (tocilizumab), Veklury (remdesivir), Olumiant (baricitinib), Lagevrio (molnupiravir), and Paxlovid (nirmatrelvir and ritonavir), has gained U.S. FDA approval for the treatment of COVID-19 [[49], [50], [51], [52]]. The efficacy of these antivirals largely depends on their ability to inhibit viral replication and transcription, hence reducing the severity of SARS-CoV-2 infections. In the viral life cycle, ACE2 acts as a crucial receptor facilitating the entry of SARS-CoV-2 into host cells. Therefore, therapeutic strategies focusing on blocking ACE2-mediated virus binding offers an alternative line of defense prior to viral replication. This concept has driven the proposal of several approaches that aim to interrupt virus binding, such as depleting the ACE2 pool through the use of recombinant soluble ACE2, or the administration of neutralizing antibodies against ACE2 [18]. However, therapeutics leveraging ACE2 come with potential pitfalls, as they may induce side effects and lead to inconsistent outcomes considering the activation or suppression of ACE2's physiological functions. Moreover, other pharmacological strategies have been proposed to mitigate SARS-CoV-2 infection by targeting different aspects of viral invasion, such as inhibiting membrane fusion through the prevention of clathrin-dependent endosomal fusion or reducing endocytosis, and suppressing mTORC1 activity to curtail autophagy and RNA transcription [53,54]. These multifaceted approaches underscore the necessity for continuous therapeutic advancements in COVID-19 management.

In this investigation, we endeavored to elucidate the potential of utilizing spike decoy peptides as an alternative strategy to impede the spike-ACE2 interaction, a key process for SARS-CoV-2 to establish infection. The rationale that underpins our work is that SARS-CoV-2, although mutating rapidly, is invariably selected to bind to the host ACE2 receptor, which is highly expressed across various host tissues including oral epithelium, respiratory tract, alveoli, salivary glands, intestinal cells, renal cells, and cardiac cells [14,[55], [56], [57]]. Our postulation is strongly supported by several mechanistically similar studies that have successfully employed ACE2 decoy to neutralize viral particles [[58], [59], [60]]. In consideration of pharmaceutical synthesis, the production of these small peptides is relatively straightforward, enabling swift large-scale manufacture and dynamic adjustment in response to the evolution of variant strains exhibiting elevated ACE2 affinity. Due to their comparatively smaller size than other biological macromolecules such as antibodies, they can easily diffuse across cell surfaces, penetrate interaction sites, and effectively obstruct ACE2. Accordingly, such peptides have been variably formulated as inhalable reagents, oral topical solutions, nasal sprays, and for direct oral intake. The therapeutic versatility of peptide decoys extends beyond the scope of our study, with existing proposals advocating their application in cancer treatment strategies involving host cell surface receptors, exemplified by Human Epidermal Growth Factor 2 (HER2) in breast cancer [61]. Finally, further evaluation using *in vivo* trials, as well as assessments of the pharmacokinetic properties, are needed to finetune these of spike decoy peptides as prophylactic agents against SARS-CoV-2 infections.

## Ethics statement

Review and approval by an ethics committee was not needed for this study because no human or animal subjects were used in the experiments.

## Data availability statement

Data available within the article or its supplementary materials. Raw data available for download from NYCU Dataverse (https://dataverse.lib.nycu.edu.tw/).

## CRediT authorship contribution statement

**Lai-Keng Loi:** Writing – review & editing, Writing – original draft, Visualization, Validation, Software, Resources, Project administration, Methodology, Investigation, Formal analysis, Data curation, Conceptualization. **Cheng-Chieh Yang:** Writing – review & editing, Writing – original draft, Validation, Supervision, Resources, Project administration, Investigation, Funding acquisition, Formal analysis, Data curation, Conceptualization. **Yu-Cheng Lin:** Writing – review & editing, Writing – original draft, Validation, Supervision, Project administration, Funding acquisition, Data curation. **Yee-Fun Su:** Software, Investigation, Conceptualization. **Yi-Chen Juan:** Software, Investigation, Conceptualization. **Yi-Hsin Chen:** Software, Resources, Methodology, Investigation, Formal analysis, Data curation, Conceptualization. **Hsiu-Chuan Chang:** Validation, Methodology, Investigation, Conceptualization.

## Declaration of generative AI and AI-assisted technologies in the writing process

During the preparation of this work the authors used ChatGPT (GPT-4 May 24 Version) for grammatical correction and semantic recommendation. After using ChatGPT, the authors reviewed and edited the content as needed and take full responsibility for the content of the publication.

## Declaration of competing interest

The authors declare that they have no known competing financial interests or personal relationships that could have appeared to influence the work reported in this paper.

## Appendix A Supplementary data

Multimedia component 1 **Figure S1**. Western blot control and confocal fluorescence microscopic images of ACE2. (A) Western blot showing the molecular size of ACE2 at 110 kDa; par., parental; VA; OE, over-expression. (B) confocal images showing ACE2 (green) localized on the cell membrane. Multimedia component 1

Multimedia component 2 **Figure S2**. Anti-ACE2 antibody blocks VRBMP binding to oral keratinocytes. (A) Confocal fluorescence microscopic images showing NOK cells incubated in media containing different concentrations of anti-ACE2 antibody for 30 min and then shifted to 50 μg/ml of VRBMP-1 for 24 h. ACE2, DAPI, and VRBMP-1 are shown in red, blue, and light gray, respectively. Scale bars indicate 20 μm. (B) Quantitative analysis of the average VRBMP signal Data are presented as mean ± SEM (n = 15) with statistical analysis using unpaired *t*-tests with equal variance (****p* < 0.001, *****p* < 0.0001). Multimedia component 2

Multimedia component 3 **Figure S3**. ELISA shows DP-3 peptide and DIZE inhibits RBD-ACE2 interaction. (A) Schematics of the ELISA procedure which measures the interaction between ACE2 and RBD. Dose-dependent inhibitory effect of DP-3 peptide (B, C) and DIZE (D); each panel shows an independent assay. Error bars indicate STD (n = 2). *P* values (**p* < 0.05) were determined by unpaired *t*-test with equal variance. Multimedia component 3
