# Supplementary figures and images for: Decoy peptides effectively inhibit the binding of SARS-CoV-2 to ACE2 on oral epithelial cells
Source: Heliyon. 2023 Nov 20;9(12):e22614. doi: 10.1016/j.heliyon.2023.e22614 (PMC10724569; doi:10.1016/j.heliyon.2023.e22614)

**A**

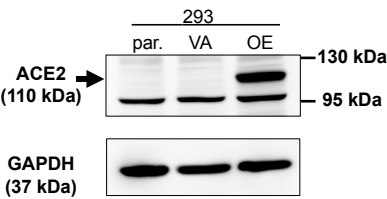

**B**

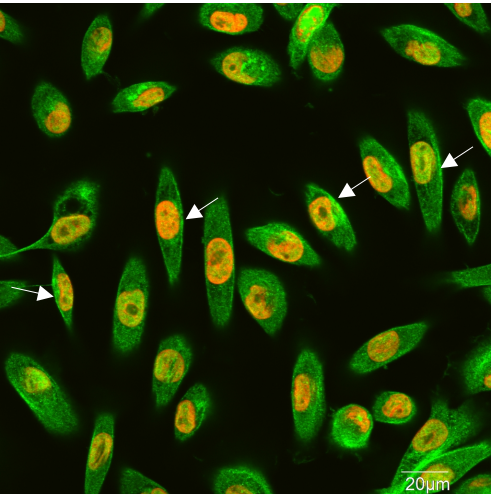

Supplement: Multimedia component 1 — Figure S1. Western blot control and confocal fluorescence microscopic images of ACE2. (A) Western blot showing the molecular size of ACE2 at 110 kDa; par., parental; VA; OE, over-expression. (B) confocal images showing ACE2 (green) localized on the cell membrane. [file mmc1.pdf]

**A**

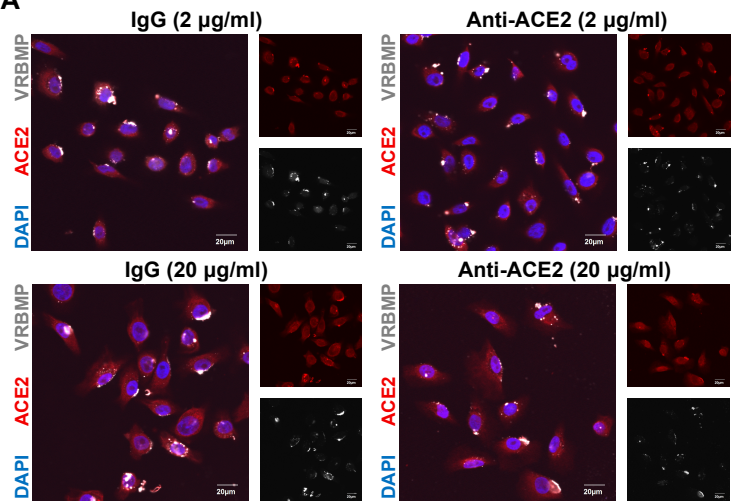

**B**

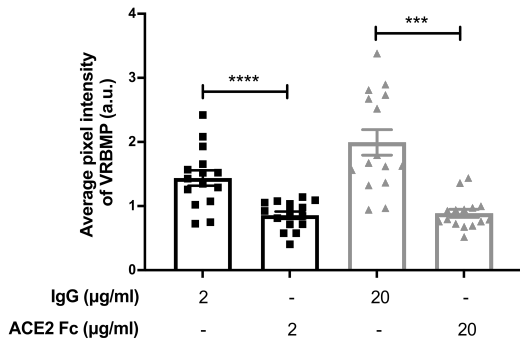

Supplement: Multimedia component 2 — Figure S2. Anti-ACE2 antibody blocks VRBMP binding to oral keratinocytes. (A) Confocal fluorescence microscopic images showing NOK cells incubated in media containing different concentrations of anti-ACE2 antibody for 30 min and then shifted to 50 μg/ml of VRBMP-1 for 24 h. ACE2, DAPI, and VRBMP-1 are shown in red, blue, and light gray, respectively. Scale bars indicate 20 μm. (B) Quantitative analysis of the average VRBMP signal Data are presented as mean ± SEM (n = 15) with statistical analysis using unpaired t-tests with equal variance (***p < 0.001, ****p < 0.0001). [file mmc2.pdf]

**A**

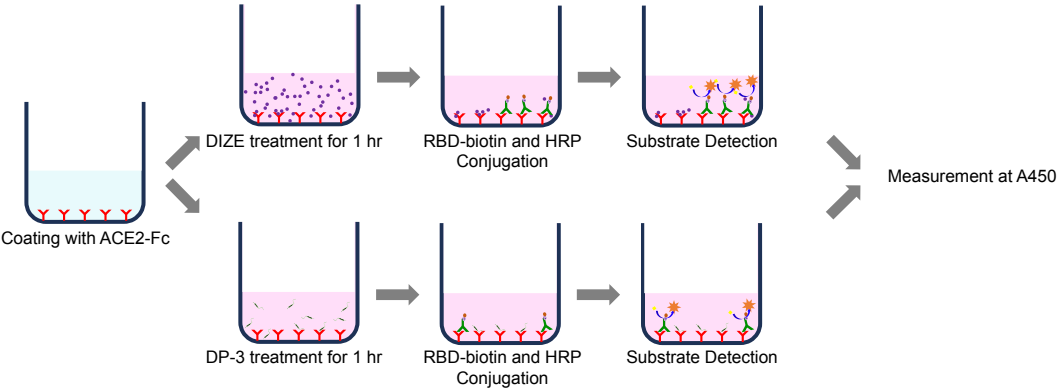

**B**

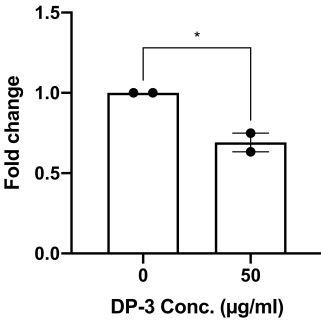

**C**

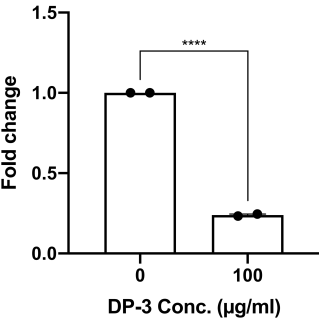

**D**

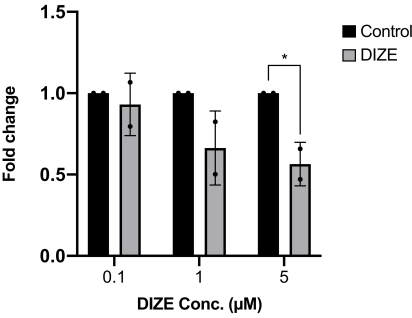

Supplement: Multimedia component 3 — Figure S3. ELISA shows DP-3 peptide and DIZE inhibits RBD-ACE2 interaction. (A) Schematics of the ELISA procedure which measures the interaction between ACE2 and RBD. Dose-dependent inhibitory effect of DP-3 peptide (B, C) and DIZE (D); each panel shows an independent assay. Error bars indicate STD (n = 2). P values (*p < 0.05) were determined by unpaired t-test with equal variance. [file mmc3.pdf]
